# Rat volatiles as an attractant source for the Asian tiger mosquito, *Aedes albopictus*

**DOI:** 10.1038/s41598-020-61925-z

**Published:** 2020-03-20

**Authors:** Edvin Díaz-Santiz, Julio C. Rojas, Mauricio Casas-Martínez, Leopoldo Cruz-López, Edi A. Malo

**Affiliations:** 1 Grupo de Ecología de Artrópodos y Manejo de Plagas, Departamento de Agricultura, Sociedad y Ambiente, El Colegio de la Frontera Sur (ECOSUR), Carretera Antiguo Aeropuerto km 2.5, Tapachula, Chiapas 30700 México; 2 Centro Regional de Investigación en Salud Pública (CRISP), Instituto Nacional de Salud Pública, 4ª Avenida Norte y 19 Calle Poniente s/n, Colonia Centro, Tapachula, Chiapas 30700 México

**Keywords:** Chemical biology, Ecology

## Abstract

*Aedes albopictus* is a vector of dengue, chikungunya, and dirofilariasis. Volatile compounds are crucial for mosquitoes to locate their hosts. This knowledge has allowed the identification of attractants derived from human odours for highly anthropophilic mosquito species. In this study, we used rats as a experimental model to identify potential attractants for host-seeking *Ae. albopictus* females. Porapak Q extracts from immature female rats were more attractive to *Ae. albopictus* females than those from mature and pregnant females, and males. Phenol, 4-methylphenol, 4-ethylphenol, and indole were identified compounds in male, immature, mature, and pregnant female extracts. There were quantitative differences in these compounds among the extracts that likely explain the discrepancy in their attractiveness. *Ae. albopictus* females were not attracted to the single compounds when was compared with the four-component blend. However, the binary blend of 4-methylphenol  + 4-ethylphenol and the tertiary blend of 4-methylphenol + 4-ethylphenol + indole were as attractive as the four-component blend. In the field trials, BGS traps baited with the tertiary or quaternary blends caught more *Ae. albopictus* females and males than BGS traps without lures. This is the first laboratory and field study to identify compounds that mediate the attraction of *Ae*. *albopictus* to one of its hosts.

## Introduction

*Aedes albopictus* (Diptera: Culicidae) is a mosquito native to Southeast Asia, however is a highly invasive mosquito species and is difficult to control^1,2^. This mosquito species is considered a secondary vector of dengue, other arboviruses and canine dirofilarisis^2,3^. Recently, *Ae. albopictus* has also been incriminated as a primary vector in epidemics of chikungunya fever (CHIK) in several countries bordering the Indian Ocean, Central Africa and Europe^4,5^. In addition, *Ae. albopictus* has shown a rapid geographical spread in recent decades^6^, as it has high genetic, physiological and ecological plasticity^7^. *Ae. albopictus* is frequently found in suburban and rural areas where open spaces with vegetation predominate. Several studies have reported that this mosquito species feeds on a great number of hosts, such as mammals, birds, amphibians, and reptiles^8–11^, suggesting that *Ae. albopictus* is an opportunistic mosquito and possibly a vector of zoonotic and human arboviruses.

The host-seeking behaviour of mosquitoes is a complex process that involves the use of chemical and physical cues emitted by the hosts^12,13^. This knowledge has allowed the identification of different attractant blends derived from human odours for highly anthropophilic mosquito species, including *Anopheles* spp., *Culex quinquefasciatus*, and *Aedes aegypti*^12,14–19^. Some of these blends have been evaluated with *Ae. albopictus* mosquitoes. For example, BG-Sentinel (BGS) traps baited with a BG-lure (ammonia, lactic acid and caproic acid), octenol and CO_2_ captured more *Ae. albopictus* mosquitoes than CDC light traps and gravid traps^20^. Further, it was found that the L-lactic acid in dichloromethane was highly attractive to this mosquito species^21^. Recently, a new commercial formulation, BG-Sweet scent (based on lactic acid), showed that it is as efficient as the BG-lure for capturing *Ae*. *albopictus* mosquitoes^22^. As mentioned previously, most of the lures have been formulated using human volatile compounds. However, the fact that *Ae. albopictus* is an opportunistic mosquito opens the possibility of evaluating other hosts for attractive volatile sources. Indeed, field studies showed that BGS traps baited with mice (*Mus musculus*) increased the capture of *Ae. albopictus* males and females compared to BGS traps without mice^23,24^. In addition, it was reported that BGS traps baited with 3 mice (2 females and 1 male) captured more mosquitoes than BGS traps baited with a BG-lure or 1 male mouse^25^. The latter authors mentioned that the odours from mouse excretions (such as urine and faeces), in addition to skin odours, CO_2_ and heat from the mice themselves, could have been responsible for the high mosquito attraction to the traps. Le Goff *et al*.^25^ also speculated that the presence of female and male mice in the trap could have induced some behaviour that increased the odours that affected mosquito attraction.

A previous study has reported that *Ae. albopictus* females were able to feed on rat (*Rattus novergicus*) blood when this was offered in laboratory^8^. Recently, mosquito blood sampled from neighbourhoods in Baltimore, MD, USA has shown that *R. novergicus* was the most often detected host in *A*. *albopictus*^26^. Therefore, in this study, we used laboratory rats, *R*. *novergicus*, as an experimental model to search for potential attractants for *Ae. albopictus* females. First, we evaluated the attraction of *Ae*. *albopictus* females to volatile extracts from laboratory rats of different sexes and physiological stages (immature, mature, or pregnant). Second, we identified the volatile compounds present in the attractive extracts by gas chromatography-mass spectrometry. Finally, we evaluated the biological activity of the identified compounds and their blends in laboratory bioassays and field trials. This report constitutes the first study to identify compounds that mediate the attraction of *Ae*. *albopictus* to one of its vertebrate hosts.

## results

### Behavioural responses of *Ae. albopictus* mosquitoes to immature female or male rat extracts

**Figure 1:**
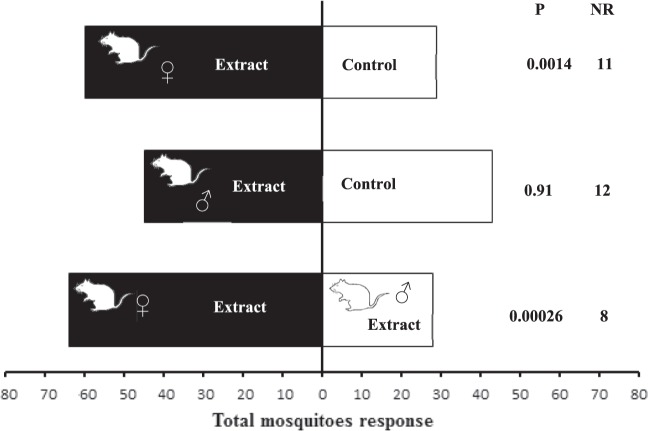
Attraction of *Aedes albopictus* immature female or male rat extracts. FR (female rat), MR (male rat), Control (filter paper strip loaded with dichloromethane). N = 20 replicates by treatment. NR = non-responding.

*Ae. albopictus* females were more attracted to immature female rat extracts than the solvent control (χ² = 10.11, Df = 1, P = 0.00147, *n *= 20) (Fig. 1). In contrast, mosquito females did not show any preference for the immature male rat extracts or the solvent control (χ² = 0.01, Df = 1, P = 0.915, *n *= 20). In addition, *Ae. albopictus* females were more attracted to immature female rat extracts than immature male rat extracts (χ² = 13.31, Df = 1, P = 0.00026, *n *= 20). These results suggest that rat gender affected the attraction of mosquito females.

### Behavioural responses of *Ae. albopictus* mosquitoes to extracts from rat females in different physiological stages

**Figure 2:**
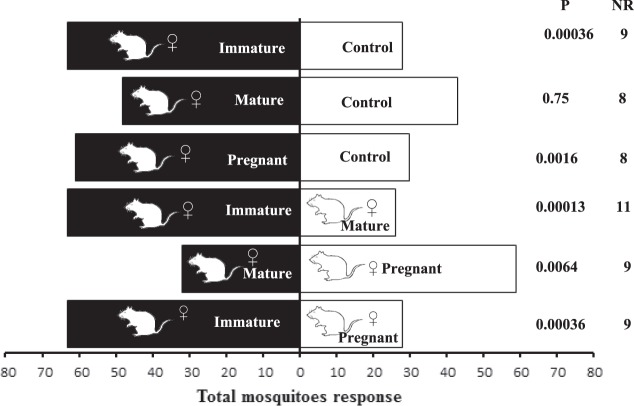
Attraction of *Aedes albopictus* females to rat extracts in different physiological stages. IFR (Immature Female Rat), MFR (Mature Female Rat), PFR (Pregnant Female Rat). N = 20 replicates by treatment. NR = non-responding.

*Ae. albopictus* females were more attracted to immature female rat extracts than the control (χ² = 12.70, Df = 1, P = 0.00036, *n *= 20) (Fig. 2). Similar results were observed with pregnant female rat extracts versus the control (χ² = 9.89, Df = 1, P = 0.00166, *n *= 20). In contrast, *Ae. albopictus* females did not show increased attraction to mature female rat extracts compared with the solvent control (χ² = 0.97, Df = 1, P = 0.754, *n *= 20) (Fig. 2). In addition, we observed that *Ae. albopictus* females were more attracted to immature female rat extracts than the mature female rat extracts (χ² = 14.56, Df = 1, P = 0.00013, *n *= 20) (Fig. 2). Similar results were found when pregnant female rat extracts were tested against mature female rat extracts (χ² = 7.42, Df = 1, P = 0.00642, *n *= 20). However, *Ae. albopictus* females were more attracted to immature female rat extracts than pregnant female rat extracts (χ² = 12.70, Df = 1, P = 0.00036, *n *= 20).

### Chemical analysis

**Figure 3:**
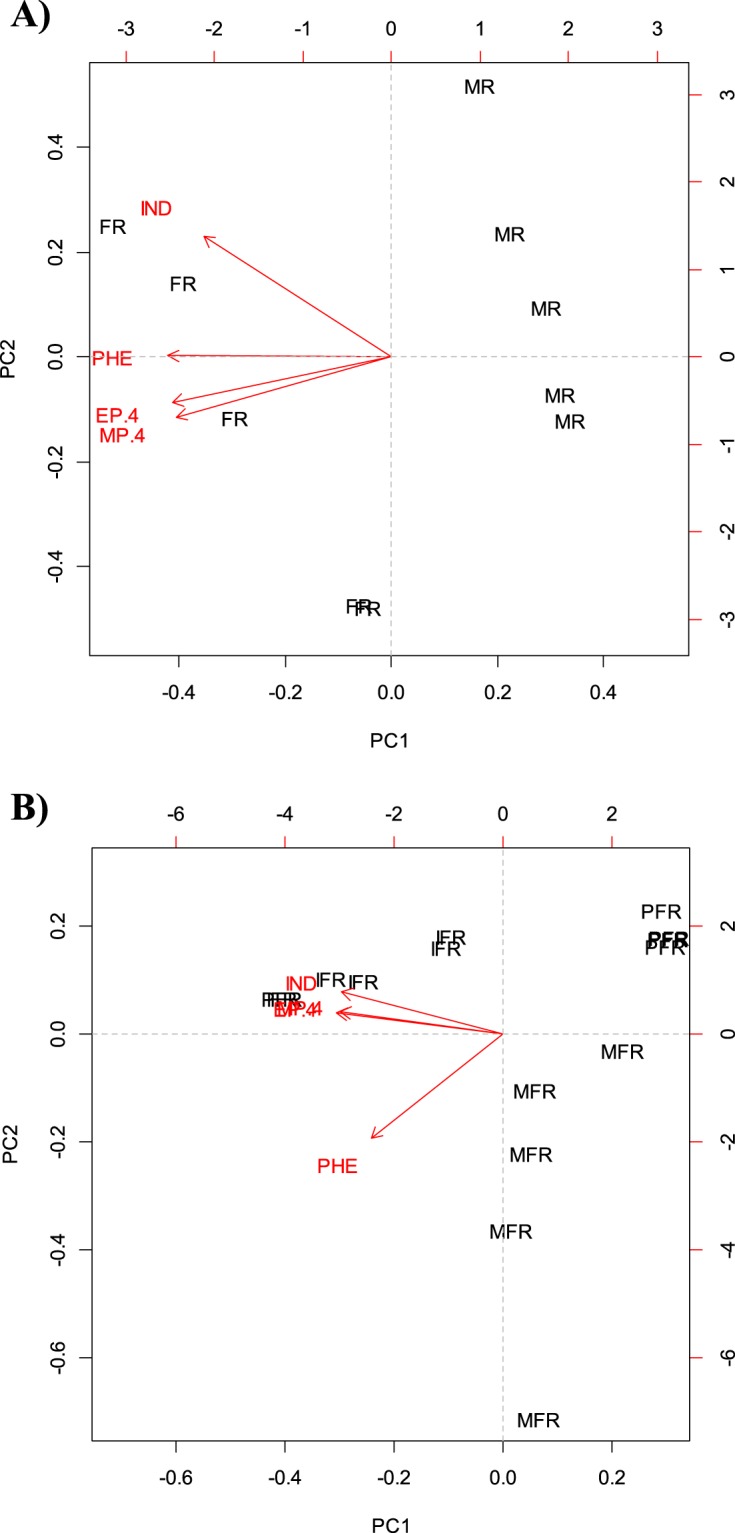
Principal components analysis of volatiles emitted by rat extracts (sex and different physiological stages). (**A**) Volatiles emitted by male and female rat extracts. The graph shows the separation of the compounds present in the two extracts with respect to the concentration in which they were found (PC1 = 89.47, PC2 = 10.32, Total = 99.79). MR = male rat extract, FR = female rat extract. Compounds phenol (PHE), 4-methylphenol (MP.4), 4-ethylphenol (EP.4), indole (IND). (**B**) Volatiles emitted by female rat extracts of different physiological stages. The graph shows the separation of the compounds present in the extracts with respect to the concentration in which they were captured (PC1 = 46.26, PC2 = 34.18, Total = 80.44). IFR = Immature Female Rat extract, MFR = Mature Female Rat extract, PFR = Pregnant Female Rat extract. Compounds phenol (PHE), 4-methylphenol (MP.4), 4-ethylphenol (EP.4), indole (IND).

All rat extracts consistently contained four compounds that were identified by GC-MS as phenol, 4-methylphenol, 4-ethylphenol, and indole (Supplementary Figs. S1 and S2). The main component was 4-methylphenol, followed by 4-ethylphenol, phenol, and indole in the rat extracts. However, these compounds were found in different proportions in the male and female rat extracts and among immature, mature, and pregnant rat female extracts (Supplementary Table S1). The principal components analysis (PCA) of the proportion of the volatiles present in the male and female rat extracts showed a clear separation between groups (Fig. 3A). The results of PC1 and PC2 explained 99.79% of the variance in the data. The PCA of the proportion of the volatiles in extracts from immature, mature, and pregnant rat females showed a clear separation among groups (Fig. 3B). The results of PC1 and PC2 explained 80.44% of the variance in the data.

### Behavioural responses of *Ae. albopictus* mosquitoes to synthetic compounds

**Figure 4:**
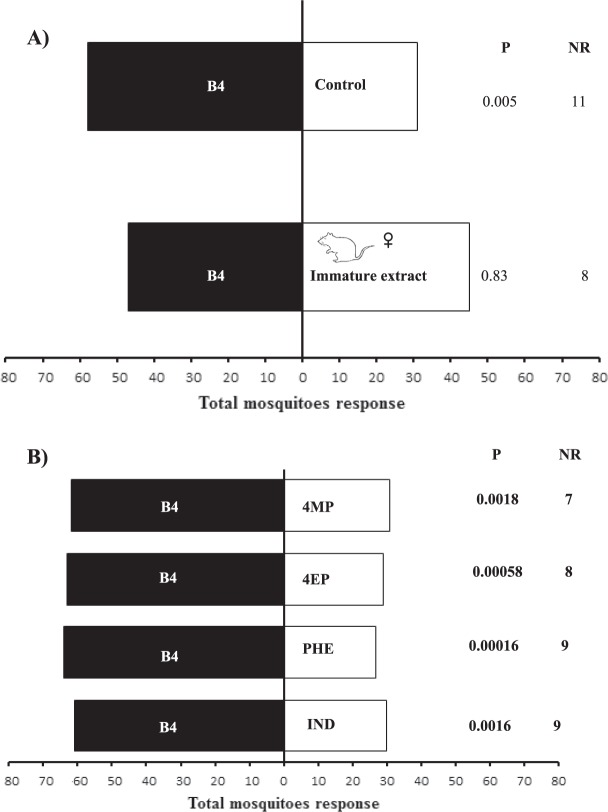
Attraction of *Aedes albopictus* females to synthetic blends. (**A)** Attraction of *Aedes albopictus* females to synthetic blend (B4) compared to control, immature female rat extracts IFR (Immature Female Rat). B4 = phenol, 4-methylphenol, 4-ethylphenol and indole. (**B)** Relative attraction of *Aedes albopictus* females to single compound compared to synthetic blend (B4). B4 = phenol, 4-methylphenol, 4-ethylphenol and indole. 4MP = 4-methylphenol, 4EP = 4-ethylphenol, IND = Indole, PHE = Phenol. N = 20 replicates by treatment. NR = non-responding.

*Ae. albopictus* females were more attracted to the four-compound synthetic blend (B4) than the solvent control (χ² = 7.59, Df = 1, P = 0.00585, *n *= 20). *Ae. albopictus* females did not show a preference for B4 or the immature female rat extract (χ² = 0.04, Df = 1, P = 0.8339, *n *= 20) (Fig. 4A). Mosquito females were more attracted to B4 than to phenol (χ² = 114.24, Df = 1, P = 0.00016, *n *= 20), 4-methylphenol (χ² = 9.67, Df = 1, P = 0.00186, *n *= 20), 4-ethylphenol (χ² = 11.83, Df = 1, P = 0.00058, *n *= 20), or indole (χ² = 9.89, Df = 1, P = 0.00166, *n *= 20) when each compound was tested individually (Fig. 4B).

**Figure 5:**
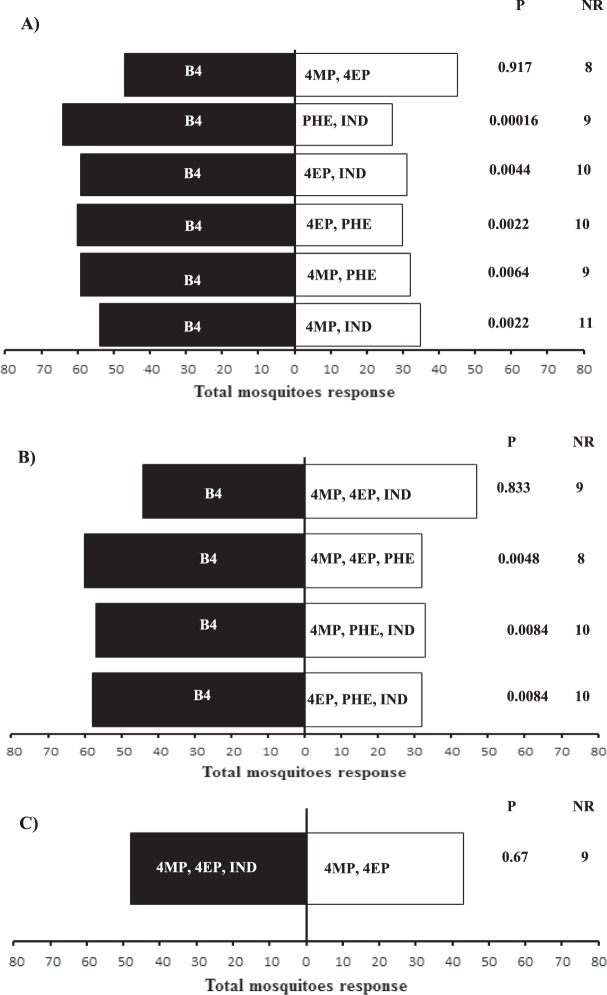
Attraction of *Aedes albopictus* females to synthetic blend (B4) compared to binary blends. B4 = phenol, 4-methylphenol, 4-ethylphenol and indole. 4MP = 4-methylphenol, 4EP = 4-ethylphenol, IND = Indole, PHE = Phenol. (**B)** Attraction of *Aedes albopictus* females to synthetic blend (B4) compared to tertiary blends. B4 = phenol, 4-methylphenol, 4-ethylphenol and indole. 4MP = 4-methylphenol, 4EP = 4-ethylphenol, IND = Indole, PHE = Phenol. N = 20 replicates by treatment. (**C)** Attraction of *Aedes albopictus* females to tertiary blend (4MP, 4EP, and indole) compared to binary blend (4MP, 4EP). 4MP = 4-methylphenol, 4EP = 4-ethylphenol, IND = Indole, PHE = Phenol. N = 20 replicates by treatment. NR = non-responding.

When the biological activity of the binary blends was evaluated, we observed that *Ae. albopictus* females were equally attracted to the 4-methylphenol + 4-ethylphenol blend and B4 (χ² = 0.01, Df = 1, P = 0.917, *n *= 20) (Fig. 5A). However, *Ae. albopictus* females were more attracted to B4 than the phenol + indole blend (χ² = 14.24, Df = 1, P = 0.00016, *n *= 20), 4-ethylphenol + indole blend (χ² = 8.1, Df = 1, P = 0.0044, *n *= 20), 4-ethylphenol + phenol blend (χ² = 9.34, Df = 1, P = 0.0022, *n *= 20), 4-methylphenol +  phenol blend (χ² = 7.42, Df = 1, P = 0.0064, *n *= 20), and 4-methylphenol + indole blend (χ² = 9.34, Df = 1, P = 0.0022, *n *= 20) (Fig. 5A).

When the biological activity of the tertiary blends was evaluated, we found that *Ae. albopictus* females were equally attracted to B4 and the 4-methylphenol + 4-ethylphenol + indole blend (χ² = 0.04, Df = 1, P = 0.8339, *n *= 20) (Fig. 5B). In contrast, the mosquitoes were more attracted to B4 than the phenol + 4-methylphenol + 4-ethylphenol blend (χ² = 7.92, Df = 1, P = 0.00487, *n *= 20), phenol + 4-methylphenol + indole blend (χ² = 6.94, Df = 1, P = 0.00840, *n *= 20), and phenol + 4-ethylphenol + indole blend (χ² = 6.94, Df = 1, P = 0.00840, *n *= 20) (Fig. 5B).

When we compared the binary blend (4-methylphenol + 4-ethylphenol) and tertiary blend (4-methylphenol + 4-ethylphenol + indole), which showed the best performance in previous bioassays, we found that the mosquito females were equally attracted to both blends (χ² = 0.17, Df = 1, P = 0.675, *n *= 20) (Fig. 5C).

### Field trials

A total of 1310 adult mosquitoes were collected in the Jardin cemetery during the field study, including 1023 *Ae. albopictus* (542 females and 481 males), and 287 *Ae. aegypti* (80 females and 207 males). The number of *Ae. albopictus* collected by the BGS traps were as follows: control traps (BGS without lures) captured 133 *Ae. albopictus* mosquitoes (13%) (73 females and 60 males), traps baited with the binary blend (4-methylphenol + 4-ethylphenol) caught 193 tiger mosquitoes (18.8%) (118 females and 75 males), traps baited with the 4-methylphenol + 4-ethylphenol and indole blend captured 360 *Ae. albopictus* mosquitoes(35.3%) (155 females and 205 males) and traps baited with the quaternary blend (phenol + 4-methylphenol + 4-ethylphenol and indole) caught 337 *Ae. albopictus* mosquitoes (32.9%) (196 females and 141 males).

The total number of *Ae. albopictus* mosquitoes caught varied significantly among the treatments (F = 9.9; Df = 3,21; P = 0.00028). However, the observation dates (F = 1.07; Df = 3,21; P = 0.382), site (F = 1.04; Df = 3,21; P = 0.392) and replicates (F = 3.72; Df = 3,21; P = 0.0671) did not affect the capture of mosquitoes. Also, the number of *Ae. albopictus* females caught varied significantly among the treatments (F = 7.07; Df = 3, 21; P = 0.0018) and replicates (F = 6 0.69; Df. = 3, 21; P = 0.017). In contrast, the observation dates (F = 1.14; Df = 3, 21; P = 0.352) and site (F = 1.80; Df = 3, 21; P = 0.177) did not affect the capture. Similarly, the number of *Ae. albopictus* males caught varied significantly among the treatments (F = 7.2; Df = 3, 21; P = 0.0016). But, the observation dates (F = 0.49; Df = 3, 21; P = 0.68) site (F = 0.87; Df = 3, 21; P = 0.467) and the replicates (F = 0.44; Df = 3, 21; P = 0.0513) did not affect the capture.

**Figure 6:**
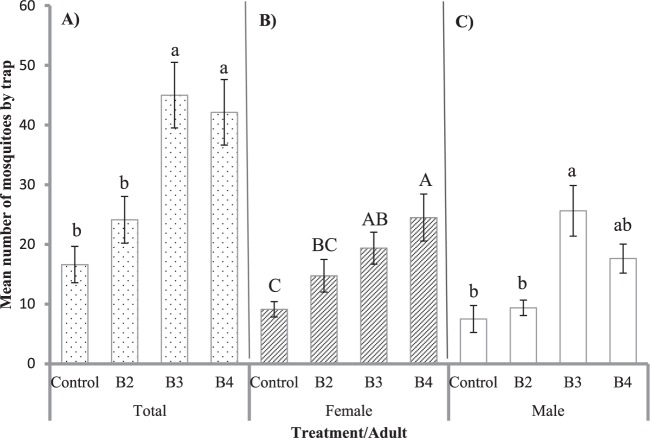
Mean number (±EE) of *Aedes albopictus* capture with BGS-traps baited with the blends attractants in the field. (**A**) Total capture (males and females) *Aedes albopictus*. (**B**) Capture of *Ae. albopictus* females. (**C**) Capture of *Ae. albopictus* males. Bars represent the mean values (n = 8). B2 = 4-methylphenol, 4-ethylphenol; B3 = 4-methylphenol, 4-ethylphenol and indole; B4 = phenol, 4-methylphenol, 4-ethylphenol and indole. Treatments with the same letter are not significantly different.

The traps baited with the tertiary (4-methylphenol + 4-ethylphenol and indole) and quaternary (phenol + 4-methylphenol + 4-ethylphenol and indole) blends captured more total mosquitoes (Fig. 6A) and females (Fig. 6B) than the traps baited with the binary blends (4-m ethylphenol + 4-ethylphenol) and the control traps. The traps baited with the tertiary blend (4-methylphenol + 4-ethylphenol and indole) captured more males than those caught by traps baited with the binary blend (4-methylphenol + 4-ethylphenol) and control traps. The traps baited with the quaternary blend (phenol + 4-methylphenol + 4-ethylphenol and indole) captured a similar number of males as those baited with the tertiary and binary blends and the control traps (Fig. 6C). The parity rate of *Ae. albopictus* females caught by traps baited with the tertiary (4-methylphenol + 4-ethylphenol + indole) and quaternary (phenol + 4-methylphenol + 4-ethylphenol + indole) blends were 30 and 39.4%, respectively.

## discussion

In this study, we used rats as an experimental model to search for attractants for the Asian tiger mosquito. This study is pioneering in identifying volatile compounds from non-humans that mediate the host-seeking process of *Ae. albopictus* females. Our results suggest that mosquito females discriminated between sexes and among immature, mature and pregnant females based on the difference in proportions of the compounds emitted by rats. The laboratory test showed that B2 (4-methylphenol + 4-ethylphenol) was just as attractive as B3 (4-methylphenol + 4-ethylphenol + indole) and B4 (4-methylphenol + 4-ethylphenol + indole + phenol). However, a field test showed that B3 or B4 were more attractive to *Ae. albopictus* females than B2. Previous studies searching for attractants have used compounds derived from human odours that influence the attraction of other mosquito species^23,24,27,28^. For instance, it has been recently reported that a blend of five compounds derived from human sweat, previously found as an attractant for *Aedes aegypti*, was slightly more efficient in attracting *Ae*. *albopictus* than the BG-lure^28^.

In our study, we found that rat sex affected the attraction of *Ae. albopictus* females. The contrast in the attraction of *Ae*. *albopictus* to female and male rats may be explained if there is a different level of susceptibility to a putative infection between sexes. For instance, some authors suggest that male mammals are more susceptible to infection by parasites or pathogens than females, attributing this to the fact that females generally have increased immune responses compared to males^29,30^. However, it has also been observed that male mice (*Mus musculus*) are less susceptible than female mice to several parasites, including *Babesia microti*, *Toxoplasma gondii, Schistosoma manson i*, and *Taenia crassiceps*^31–34^. This sexual difference in the response to certain pathogens is not well defined, but it has been suggested that the difference in the host-pathogen interaction is possibly due to the host endocrine system^35^. Alternatively, *Ae*. *albopictus* preference for the females over males might be due to the fact that the blood characteristics (e.g., nutrients, hormones) differ between sexes and this difference could affect mosquito fitness. Previously, we have found that *Ae*. *albopictus* mosquitoes fed on female rats have greater fecundity than those fed on males (unpublished data).

We also observed that *Ae*. *albopictus* females were more attracted to the volatiles from immature female rats compared to mature and pregnant females, suggesting that the physiological stage of the female rats affects the attraction of the mosquitoes. It is possible that the hormonal changes experienced by rats during their life cycle may affect the production of compounds emitted by female rats and influence the attraction of mosquitoes. The odours of most female mammalian species probably vary according to their reproductive stages^36^. For example, alterations in the relationship between progesterone and oestrogen at the end of pregnancy indicate that these hormones play an important role in maternal behaviour. In rats, the highest levels of progesterone in the blood were present at 14 and 15 days of gestation and decreased from day 19. Oestrogen began to increase on day 16, reaching its highest level on day 22^37^. The chemical components in urine may also vary with the oestrus cycle^38,39^. Moreover, volatile urinary metabolites have been analysed in the puberty stage, and similarities have been observed in the compounds present in the excretions with respect to the different periods of pregnancy and lactation^40^. Previous studies have shown that pregnant women are more attractive to *Anopheles gambiae* mosquitoes than non-pregnant women^41,42^. However, to our knowledge the responsible compounds of this differential attraction of mosquito to pregnant and non-pregnant women are not yet known.

In this study, we determined that the attractive compounds were found in higher concentration in the female rat extracts compared with the male rat extracts. This finding is different from that reported by Zhang *et al*.^43^, who observed that phenol, 4-methylphenol, and 4-ethylphenol were in higher amounts in the male extracts than in female rat extracts. The difference between both studies is possibly due to the different volatile collection techniques used. Zhang *et al*.^43^ collected the volatiles from urine diluted in dichloromethane, while we sampled the volatile compounds using the dynamic headspace technique. For example, it is known that the proportion of insect pheromone components emitted in the effluvia may differ noticeably from that found in the glands^44,45^.

The compounds identified in our work have been reported as components of excretions in rodents^36,46^. Rats and mice, voided urine, including volatile compounds derived from urine and discharged from preputial glands, serves as the major source of odour^45–48^. However, the phenols found in the present study also may be byproducts of the microbial activity present in rats. Previously, it has been reported that 4-methylphenol is derived from the bacterial degradation of tryptophan and tyrosine^49,50^. Likewise, 4-methylphenol has been identified in excretions from bovines^51^ as well as human sweat^52^, but its source is unknown. Human blood is rich in volatile aromatic compounds, including 4-ethylphenol, phenyl acetic acid, and benzoic acid^53^. Moreover, phenol has been identified as having a human-like smell^16^. These compounds are used as semiochemicals for recognition and as indicators of sex, health condition, age, and reproductive stage in the social system of mice^54,55^, possibly some of these chemical signals are used by mosquitoes as cues during the host-seeking.

The compounds identified in this study previously have shown to play a role in the attraction of mosquito females^56,57^. For instance, phenols are compounds present in natural oviposition sites^57^. In addition, phenol, 4-methylphenol, 4-ethylphenol, indole and 3-methylindole were identified in fermented natural organic infusions as cues responsible for eliciting oviposition in *Aedes, Culex* and *Anopheles*, including *Ae*. *albopictus*^51,58–60^. Moreover, 4-ethylphenol and 4-methylphenol are oviposition attractants and stimulants for *Cx*. *quinquefasciatus* that acts at a short distance^61^. It has also been suggested that 4-ethylphenol is important not only for oviposition selection but also to reduce host-seeking and initiate blood feeding in *Cx. quinquefasciatus*^62^. Our results seem to suggest that *Ae*. *albopictus* females may be making parsimonious use of phenols as cues for host-seeking and for the location of oviposition sites.

The results obtained in the bioassay laboratory showed the *Ae. albopictus* females were attracted to B2, B3 and B4. However, in the field, the tertiary and quaternary blends were more attractive than the binary blends and control. These discrepancies may be explained because we used only nulliparous females (3–4 days old, mated and non-blood-fed) in laboratory bioassays, while in the field, we caught females in different reproductive stages, reflected by the parity rate. Additionally, another possible explanation is that the laboratory bioassays were performed with clean air, while in the field; the presence of background odours might have affected the mosquito attraction to the traps. In this way, we need to consider that when conducting field tests, potential hosts in the open space (e.g., humans, dogs, birds) or in lairs or natural shelters (e.g., rodents, reptiles) emit volatiles that may compete with the experimental blends. Previous studies have documented that environmental factors and other volatile compounds can alter the attractiveness of designed blends^63^.

Although all laboratory research was performed using females in order to search for an attractant for this sex, during the field experiment, males were also captured by traps baited with the different blends. In general, more females than males were captured, except with the traps baited with the tertiary blend. This suggests that the presence of indole enhanced the male attraction to this blend, but the effect of indole was reduced by the presence of phenol in the quaternary blend. Males of *Aedes* spp assembled in the vicinity of the host apparently to catch females coming to feed^64,65^. The fact that males are attracted to hosts has been previously reported in *Ae*. *albopictus* and *Ae*. *aegypti*^66^ as well for other mosquito species^67^. Also, the swarm of *Ae*. *aegypti* males is triggered by the presence of host odours^68^. Further studies are necessary to investigate the role of identified compounds on the behaviour of *Ae*. *albopictus* males. In some situations may be desirable catching males or both sexes. According to the literature, no bait attracts mosquito males as effectively as human volunteers^64^.

From a practical point of view, the fact that the same compounds may attract *Ae*. *albopictus* females of different physiological stages may be relevant for its management. In this way, traps baited with phenols can trap nulliparous and parous females searching for a host and gravid females looking for oviposition sites, thus reducing the epidemiological risk, and eventually the mosquito populations. In the future, we will test the four-component blend identified in this study against commercial blends for mosquitoes. In addition, the four-component blend combined with other attractive components will be field-tested to find better attractants for the monitoring/management of this species.

## Material and Methods

### Mosquitoes

*Aedes albopictus* females were collected from the Jardin cemetery in Tapachula, Chiapas, with an entomological net and manual aspirator. Subsequently, females were transported to the laboratory, and groups of 75 mated females were placed into rearing cages covered with fine mesh (30 × 30 × 30 cm). The females were fed with human blood using an artificial membrane feeding system^69^. The blood was collected from volunteers at Colegio de la Frontera Sur (ECOSUR) with informed consent obtained (ECOSUR ethics committee reference number CEI-D-0145/17). Seventy-two hours after feeding, an oviposition substrate consisting of a strip (5 × 40 cm) of filter paper (Whatman # 2, Sigma-Aldrich Chemicals, St. Louis, MO, USA) inside a plastic container (10 cm high × 12.5 cm i.d.) with 100 mL of water was introduced into the cages. Females were maintained at 28.9 ± 5 °C, with 72.6 ± 5.5% relative humidity and a photoperiod of 12:12 h L:D. The experiments were performed with non-blood-fed mated females at 3–4 days old belonging to the F_1_ generation from wild mosquito females.

### Experimental hosts

Laboratory rats (*Rattus novergicus*, Wistar strain) were used in this study. The rats were obtained from Experimental Multidisciplinary Laboratory and Bioterium at the Institute of Biological Sciences of the University of Sciences and Arts of Chiapas located in Tuxtla Gutiérrez, Chiapas, Mexico. The weight of the experimental rats was 250 ± 20 g. Female rats were classified according to their physiological stage as immature (6–9 weeks old), mature (10–12 weeks old), or pregnant (13–15 weeks old). We also used 8-week-old male rats. To obtain pregnant females, one female and one male were placed together in cages (60 × 40 × 40 cm) and observed until copulation occurred. We consider a pregnant female rat 13 days after copulation, which is when the bulge of the abdomen is observed. *Rattus novergicus* have gestation period of 22–23 days.

### Ethics

Animal work was conducted under approval with the ECOSUR ethics committee and fulfilling the NOM-062-ZOO 1999 about technical specifications for production, care and use of laboratory animals. Animal welfare was assessed daily. All methods were carried out in accordance with relevant guidelines and regulations and approval was obtained from the ECOSUR.

### Chemicals

Dichloromethane, phenol, 4-methylphenol, 4-ethylphenol, and indole were purchased from Fluka and Sigma-Aldrich (Toluca, Mexico), and the purity was determined by a gas chromatography-flame ionization detector (GC-FID) as >97%.

### Volatile collection

The volatile compounds were collected using a similar dynamic headspace technique described previously^45^. A rat of a given sex or physiological stage was placed inside the chamber of a glass aeration chamber (58 cm long × 18.5 cm i.d.). The volatiles were collected by airflow passage at 1.5 L/min, (previously purified by an activated carbon filter) over the rat. The volatiles were captured in a small glass column containing (20 mg) Super Q adsorbent (50–80 mesh; Water Associates, Milford, MA, USA). The volatile collection lasted 24 h, after which the volatiles were eluted from the adsorbent with 400 μl of dichloromethane (HPLC-grade) and stored in small glass vials (1 ml) at −20 °C until analysis. The volatile collection was performed at a temperature of 26 ± 2°C, with 70 ± 5% relative humidity and a photoperiod of 12:12 h (L: D). The volatile extracts were obtained from laboratory rats of different sexes (five rats per sex), as well as different physiological stages (immature, mature and pregnant) (five rats per each physiological stage).

### Behavioural responses of *Ae. albopictus* mosquitoes to the Porapak Q extracts

The responses of *Ae. albopictus* females to the rat extracts were evaluated in a Y-glass tube olfactometer (stem, 15.5 cm; arms, 11.5 cm at 45°, internal diameter, 2.5 cm). Airflow was passed at 0.5 L/min through each arm; the air was purified by activated charcoal and humidified before passing over the target and entering the olfactometer. Ten microliters of the extract or solvent (dichloromethane) was applied to a strip (1 × 1 cm) of filter paper (Whatman No. 2, Maidstone, England). The solvent was allowed to evaporate for 20 s, and then the filter paper strip with the extract was placed into one of the olfactometer’s arms. A filter paper strip loaded with dichloromethane (control) was introduced into the opposite arm. Preliminary observations showed that *Ae*. *albopictus* females performed better when evaluated in groups rather than individually (EDS personal observation). Consequently, groups of five *Ae. albopictus* females were gently introduced into the base of the “Y” tube and given 5 min to fly to the end of one of the olfactometer arms. The choice was recorded when a mosquito reached the end of the arm and remained there for the rest of the bioassay. If the mosquitoes did not reach the end of one of the two arms after 5 min, the mosquitoes were considered non-responders. After each trial, the olfactometer was disassembled, washed with water and neutral soap, soaked with acetone and dried in the oven at 120 °C for 30 min to avoid contamination. Twenty replicates were performed with *Ae. albopictus* females for each comparison. The observations were made under artificial white light (approximately 1750 lux) at 25 ± 2 °C and 60 ± 5% relative humidity. The bioassays were performed between 06:00–10:00 h, a period that corresponds to the first activity peak in *Ae*. *albopictus* in the field^66^.

### Chemical analysis

The analysis of the samples was performed in a gas chromatograph coupled to a mass spectrometer (Shimadzu GC-2010 Plus, Tokyo, Japan) equipped with a CPWAX 57CB polar capillary column (30 m by 0.25 mm i.d.). The analysis was carried out with an initial temperature programme of 40 °C for min, with an increase of 5 °C/min, until reaching a final temperature of 250 °C and remaining at this temperature for 10 min. The mass spectrum was obtained by electronic impact, and the identification of the volatile compounds was carried out by comparing the spectral data of each compound with the NIST-98 database. Subsequently, confirmation was made by comparing the retention time and its respective mass spectrum to those from authentic standards. Calibration curves using known concentrations were constructed for each compound using a gas chromatograph to determine the concentration of each compound in the extracts. The analysis was performed using the same conditions described in the CG-MS analysis.

### Behavioural responses of *Ae. albopictus* mosquitoes to synthetic compounds

Individual compounds (phenol, 4-methylphenol, 4-ethylphenol, or indole) and their binary, tertiary, and quaternary blends were dissolved in dichloromethane. The amount of each compound or blend used in the bioassays was based on the concentration of each compound in the immature female rat extracts (Supplementary Table S1). The blend was evaluated in a “Y” glass tube olfactometer as described above. Ten microliters of the test solution (e.g., single compounds or blends) or solvent (dichloromethane) was applied to filter paper. In the first experiment, we evaluated the attraction of *Ae. albopictus* females to a 4-component synthetic blend against the control or against the immature female rat extracts. In the second experiment, we evaluated the biological activity of the single compounds (phenol, 4-methylphenol, 4-ethylphenol and indole). In the third experiment, we evaluated the biological activity of the binary blends: (1) 4-methylphenol + 4-ethylphenol, (2) phenol  + indole, (3) 4-ethylphenol + indole, (4) 4-ethylphenol + phenol, (5) 4-methylphenol + phenol, and (6) 4-methylphenol + indole. In the fourth experiment, we evaluated the attractiveness of the tertiary blends: (1) 4-methylphenol + 4-ethylphenol + indole, (2) 4-methylphenol + 4-ethylphenol + phenol, (3) phenol + 4-methylphenol + indole, and (4) phenol + 4-ethylphenol + indole. The biological activities of the binary and tertiary compounds were evaluated against the 4-component blend. Finally, the most attractive binary blend was compared against the best tertiary blend.

### Field trial

The field trial was performed in the Jardin cemetery (14°53′36″N, 092°14′48″W), located at 158 masl in Tapachula, Chiapas, Mexico, from July 15-August 16, 2019. We previously recorded high populations of *Ae*. *albopictus* in this cemetery^66^. We used BG-Sentinel traps (Biogents AG, Regensburg, Germany) to evaluate the synthetic blends. The treatments were installed in the cemetery using a Latin square experimental design for controlling the variability among the different sites and the different trapping periods. The experimental design was replicated two times using the same line (site) and column (rotation). The distance between traps was 200 m. Initially, the release rate of each compound was determined to prepare the synthetic blends (Supplementary Table S2). Each treatment was loaded on rubber septa and placed inside the trap. The following synthetic blends were evaluated: (1) 4-component blend or B4 (phenol + 4-methylphenol + 4-ethylphenol + indole), (2) 3-component blend or B3 (4-methylphenol + 4-ethylphenol + indole), (3) 2-component blend or B2 (4-methylphenol + 4-ethylphenol), and (4) empty trap (control). The number of mosquitoes caught by the traps was recorded from 06:00 to 09:00 h, the period of the first biting peak of *Ae. albopictus*^66^. When the traps were emptied, they were picked up and put out again 2 days later for the next evaluation. This protocol was repeated 3 times to complete the first replicate. Ten days later, traps were again installed for the second replicate using the same experimental design. Thus, the experiment lasted for 30 days. All the collected mosquitoes were transported to the laboratory to be identified and separated by species and sex. The mosquitoes were identified using morphological characteristics^70^. Moreover, the physiological reproductive stage (nulliparous or parous) of females was determined by abdominal dissections, observing the tracheoles of the ovary under a microscope following the standard method for Diptera of medical importance^71^.

### Statistical analysis

The attractiveness of extracts or synthetic compounds was determined as the total mosquitoes that selected a stimulus in the “Y” glass tube olfactometer bioassay. The difference in attractiveness between the treatments and control was analyzed by a Generalized Linear Model (GLM) with binomial distribution with link logit function. A principal component analysis (PCA) was also carried out to determine if the extract attractiveness could be explained by the proportion of the volatile compounds present in the extracts. The number of mosquitoes in the field trial was analysed by a GLM with negative binomial distribution with link logit function. The GLM procedure was performed by using the number of *Ae. albopictus* mosquitoes collected in each trap as dependent variable, and the treatments (B4, B3, B2 and control) and sites as fixed independent variables, and observation dates (include the trap rotation) were used as factors. Multiple comparison procedures (Tukey´s HSD tests) were also performed to test significant differences in the total number of mosquitoes caught or by sex among different treatments. All analyses were performed using the R program (Version 3.5.1).

## Supplementary information

Supplementary Information.

## Data Availability

The authors declare have not conflict in providing the data if necessary, so this data are available if the Scientific Reports required.

## References

[1] Scholte EJ (2007). The Asian tiger mosquito (*Aedes albopictus*) in the Netherlands: should we worry?. Proc. Neth. Entomol. Soc. Meet..

[2] Gratz NG (2004). Critical review of the vector status of *Aedes albopictus*. Med. Vet. Entomol..

[3] Hochedez P (2006). Chikungunya infection in travelers. Emerg. Infect. Dis..

[4] Paupy C, Delatte H, Bagny L, Corbe V, Fontenille D (2009). *Aedes albopictus*, an arbovirus vector: from the darkness to the light. Microb. Infect..

[5] Pagés F (2009). *Aedes albopictus* Mosquito: The Main Vector of the 2007 Chikungunya Outbreak in Gabon. PLoS One..

[6] Benedict, M. Q., Levine, R. S., Hawley, W. A. & Lounibos, P. Spread of the tiger: global risk of invasion by the mosquito *Aedes albopictus*. *Vector Borne Zoonotic Dis.* **7**, 76–85 (2007). 10.1089/vbz.2006.0562 PMC2212601 17417960

[7] Hawley W (1988). The biology of *Aedes albopictus*. J. Am. Mosquito Contr. Assoc..

[8] Delatte Helene, Desvars Amelie, Bouétard Anthony, Bord Séverine, Gimonneau Geoffrey, Vourc'h Gwenaël, Fontenille Didier (2010). Blood-Feeding Behavior ofAedes albopictus, a Vector of Chikungunya on La Réunion. Vector-Borne and Zoonotic Diseases.

[9] Tuten H, Bridges W, Paul K, Adler P (2012). Blood-feeding ecology of mosquitoes in zoos. Med. Vet. Entomol..

[10] Faraji A (2014). Comparative host feeding patterns of the Asian tiger mosquito, *Aedes albopictus*, in urban and suburban Northeastern USA and implications for disease transmission. PLoS Negl. Trop. Dis..

[11] Sivan A, Shriram AN, Sunish IP, Vidhya PT (2015). Host-feeding pattern of *Aedes aegypti* and *Aedes albopictus* (Diptera: Culicidae) in heterogeneous landscapes of South Andaman, Andaman and Nicobar Islands, India. Parasitol Res..

[12] Takken W, Knols BG (1999). Odor-mediated behavior of Afrotropical malaria mosquitoes. Annu. Rev. Entomol..

[13] Lehane, M. J. *The biology of blood-sucking in insects*. Cambridge: Cambridge University Press; (2005).

[14] Harrington LC, Edman JD, Scott TW (2001). Why do female *Aedes aegypti* (Diptera: Culicidae) feed preferentially and frequently on human blood?. J. Med. Entomol..

[15] Bernier UR, Kline DL, Schreck CE, Yost RA, Barnard DR (2002). Chemical analysis of human skin emanations: comparison of volatiles from humans that differ in attraction of *Aedes aegypti* (Diptera: Culicidae). J. Am. Mosq. Contr. Assoc..

[16] Curran AM, Rabin SI, Prada PA, Furton KG (2005). Comparison of the volatile organic compounds present in human odor using SPME-GC/MS. J. Chem. Ecol..

[17] Curran AM, Ramirez CF, Schoon AA, Furton KG (2007). The frequency of occurrence and discriminatory power of compounds found in human scent across a population determined by SPME-GC/MS. J. Chromatogr. B..

[18] Gallagher M (2008). Analyses of volatile organic compounds from human skin. Br. J. Dermatol..

[19] Syed Z, Leal WS (2009). Acute olfactory response of Culex mosquitoes to a human- and bird-derived attractant. Proc. Natl. Acad. Sci..

[20] Farajollahi A (2009). Field efficacy of BG-Sentinel and industry-standard traps for *Aedes albopictus* (Diptera: Culicidae) and West Nile virus surveillance. J. Med. Entomol..

[21] Hao H, Sun J, Dai J (2012). Preliminary analysis of several attractants and spatial repellents for the mosquito, *Aedes albopictus* using an olfactometer. J. Insect Sci..

[22] Akaratovic KI, Kiser JP, Gordon S, Abadam CF (2017). Evaluation of the trapping performance of four biogents AG traps and two lures for the surveillance of *Aedes albopictus* and other host-seeking mosquitoes. J. Am. Mosq. Contr. Assoc..

[23] Lacroix R, Delatte H, Hue T, Dehecq JS, Reiter P (2009). Adaptation of the BG-Sentinel trap to capture male and female. Med. Vet. Entomol..

[24] Gouagna LC, Dehecq JS, Fontenille D, Dumont Y, Boyer S (2015). Seasonal variation in size estimates of *Aedes albopictus* population based on standard mark-release-recapture experiments in an urban area on Reunion Island. Acta Trop..

[25] Le Goff G (2016). Enhancement of the BG-Sentinel trap with varying number of mice for field sampling of male and female *Aedes albopictus* mosquitoes. Parasit Vectors..

[26] Goodman H, Egisi A, Fonseca DM, Leisnham PT, Ladeau SL (2018). Primary blood-hosts of mosquitoes are influenced by social and ecological conditions in a complex urban landscape. Parasit Vectors..

[27] Pombi M (2014). Field evaluation of a novel synthetic odour blend and of the synergistic role of carbon dioxide for sampling host-seeking *Aedes albopictus* adults in Rome, Italy. Parasit Vectors..

[28] Xie L (2019). Enhancing attraction of the vector mosquito *Aedes albopictus* by using a novel synthetic odorant blend. Parasit Vectors..

[29] Zuk M, McKean KA (1996). Sex differences in parasite infections: patterns and processes. Int. J. Parasitol..

[30] Klein SL (2000). The effects of hormones on sex differences in infection: from genes to behavior. Neurosci. Biobehav. Rev..

[31] Eloi-Santos S, Olsen NJ, Correa-Oliveira R, Colley DG (1992). *Schistosoma mansoni*: mortality, pathophysiology, and susceptibility differences in male and female mice. Exp. Parasitol..

[32] Larralde C, Morales J, Terrazas I, Govezensky T, Romano MC (1995). Sex hormone changes induced by the parasite lead to feminization of the male host in murine *Taenia crassiceps cysticercosis*. J. Steroid Biochem. Mol. Biol..

[33] Walker W, Roberts CW, Ferguson DJ, Jebbari H, Alexander J (1997). Innate immunity to *Toxoplasma gondii* is influenced by gender and is associated with differences in interleukin-12 and gamma interferon production. Infect. Immun..

[34] Aguilar-Delfin I, Homer MJ, Wettstein PJ, Persing DH (2001). Innate resistance to Babesia infection is influenced by genetic background and gender. Infect. Immun..

[35] Morales-Montor J (2004). Host gender in parasitic infections of mammals: an evaluation of the female host supremacy paradigm. J. Parasitol..

[36] Achiraman S, Archunan G (2006). 1-Iodo-2 methyl undecane, a putative estrus-specific urinary chemo-signal of female mouse (*Mus musculus*). Theriogenology..

[37] Rosenblatt JS, Olufowobi A, Siegel HI (1998). Effects of pregnancy hormones on maternal responsiveness, responsiveness to estrogen stimulation of maternal behavior, and the lordosis response to estrogen stimulation. Horm. Behav..

[38] O’Connell RJ, Singer AG, Stern FL, Jesmajian S, Agosta WC (1981). Cyclic variation in the concentration of sex attractant pheromone in hamster vaginal discharge. Behav. Neural. Biol..

[39] Rajanarayanan S, Archunan G (2004). Occurrence of Flehmen in male buffaloes (*Bubalusbubalis*) with special reference to estrus. Theriogenology..

[40] Novotny MV, Ma W, Wiesler D, Zidek L (1999). Positive identification of the puberty-accelerating pheromone of the house mouse: the volatile ligands associating with the major urinary protein. Proc. R Soc. B..

[41] Lindsay S (2000). Effect of pregnancy on exposure to malaria mosquitoes. Lancet..

[42] Ansell J, Hamilton KA, Pinder M, Walraven GEL, Lindsay SW (2002). Shortrange attractiveness of pregnant women to *Anopheles gambiae* mosquitoes. Trans. R. Soc. Trop. Med. Hyg..

[43] Zhang JX, Sun LX, Zhang JH, Feng ZY (2008). Sex- and gonad-affecting scent compounds and 3 male pheromones in the rat. Chem Senses..

[44] Teal PEA, Tumlinson JH, Heath RR (1986). Chemical and behavioral analyses of volatile sex pheromone components released by calling *Heliothis virescens* (F.) females (Lepidoptera: Noctuidae). J. Chem. Ecol..

[45] Heath RR, Manukian A (1992). Development and evaluation of systems to collect volatile semiochemicals from insects and plants using a charcoal-infused medium for air purification. J. Chem. Ecol..

[46] Soini HA (2009). Comparison of urinary scents of two related mouse species, *Mus spicilegus* and *Mus domesticus*. J. Chem. Ecol..

[47] Yao-Hua Z, Jian-Xu Z (2011). Urine-Derived Key Volatiles May Signal Genetic Relatedness in Male Rats. Chem. Senses..

[48] Zhang JX, Liu YJ, Zhang JH, Sun LX (2008). Dual role of preputial gland secretion and its major components in sex recognition of mice. Physiol. Behav..

[49] Elgaali H (2002). Comparison of long-chain alcohols and other volatile compounds emitted from food-borne and related gram positive and gram negative bacteria. J. Basic. Microbiol..

[50] Lindh JM, Kannaste A, Knols BGJ, Faye I, Borg-Karlson AK (2008). Oviposition responses of *Anopheles gambiae*s s. (Diptera: Culicidae) and identification of volatiles from bacteria containing species. J. Med. Entomol..

[51] Millar JG, Chaney JD, Mulla MS (1992). Identification of oviposition attractants for *Culex quinquefasciatus* from fermented Bermuda grass infusions. J. Am. Mosquito Contr. Assoc..

[52] Cork A, Park KC (1996). Identification of electrophysiologically-active compounds for the malaria mosquito, *Anopheles gambiae*, in human sweat extracts. Med. Vet. Entomol..

[53] Loke WM (2009). A metabolite profiling approach to identify biomarkers of flavonoid in take in humans. J. Nutr..

[54] Brown RE (1979). Mammalian social odors: A critical review. Adv. stud. behav..

[55] Hurst JL (2001). Individual recognition in mice mediated by major urinary proteins. Nature..

[56] Kline DL, Takken W, Wood JF, Carlson DA (1990). Field studies on the potential of butanone, carbon dioxide, honey extract, 1-octen-3-ol, L-lactic acid and phenols as attractants for mosquitoes. Med. Vet. Entomol..

[57] Cork, A. Olfactory basis of host location by mosquitoes and other hematophagous Diptera. *Ciba Foundation Symposium*. 71–88 (1996). 10.1002/9780470514948.ch7 8894291

[58] Allan SA, Kline DL (1995). Evaluation of organic infusions and synthetic compounds mediating oviposition in *Aedes albopictus* and *Aedes aegypti* (Diptera:Culicidae). J. Chem. Ecol..

[59] Poonam, S., Paily, K. P. & Balaraman, K. Oviposition attractancy of bacterial culture filtrates: response of *Culex quinquefasciatus*. *Mem. Inst. Oswaldo Cruz* **97**, 359–362 (2002). 10.1590/s0074-02762002000300015 12048566

[60] Afify A, Galizia DCG (2015). Chemosensory cues for mosquito oviposition site selection. J. Med. Entomol..

[61] Zhu F, Xu P, Barbosa RM, Choo YM, Leal WS (2013). RNAi-based demonstration of direct link between specific odorant receptors and mosquito oviposition behavior. Insect Biochem. Mol. Biol..

[62] Choo YM, Buss GK, Tan K, Leal WS (2015). Multitasking roles of mosquito labrum in oviposition and blood feeding. Front. Physiol..

[63] Van Loon JJ (2015). Mosquito attraction: crucial role of carbon dioxide in formulation of a five-component blend of human-derived volatiles. J. Chem. Ecol..

[64] Hartberg WK (1971). Observations on the mating behavior of *Aedes aegypti* in nature. Bull World Health Organ..

[65] Dieng H (2019). Sex before or after blood feeding: Mating activities of *Aedes aegypti* males under conditions of different densities and female blood feeding opportunities. J. Asia-Pacific Entomol..

[66] Casas-Martínez M (2013). A new tent trap for monitoring the daily activity of *Aedes aegypti* and *Aedes albopictus*. J. Vector Ecol..

[67] Pitts RJ, Mozuraitis R, Gauvin-Bialecki A, Lemperiere G (2014). The roles of kairomones, synomones and pheromones in the chemically-mediated behavior of male mosquitoes. Acta Trop..

[68] Cabrera M, Jaffe K (2007). An aggregation pheromone modulates lekking behavior in the vector mosquito *Aedes aegypti* (Diptera:Culicidae). J. Am. Mosquito Contr. Assoc..

[69] Nasirian H, Ladonni H (2006). Artificial blood feeding of *Anopheles stephensi* on a membrane apparatus with human whole blood. J. Am Mosquito Contr. Assoc..

[70] Savage HM, Smith GC (1995). *Aedes albopictus* y *Aedes aegypti* en las Américas: implicaciones para la transmisión de arbovirus e identificación de hembras adultas dañadas. Boletín de la Oficina Sanitaria Panamericana..

[71] Detinova, T. S. Age-grouping methods in Diptera of medical importance. WHO Geneva, Switzerland (1962). 13885800

