# Supplementary material for: Rat volatiles as an attractant source for the Asian tiger mosquito, Aedes albopictus
Source: Sci Rep. 2020 Mar 20;10:5170. doi: 10.1038/s41598-020-61925-z (PMC7083917; doi:10.1038/s41598-020-61925-z)
Supplement: Supplementary file 1 — Supplementary Information. [file 41598_2020_61925_MOESM1_ESM.docx]

**Supplementary** **Information**

**Rat volatiles as an attractant source for the Asian tiger mosquito, *Aedes albopictus***

Díaz-Santiz Edvin^1^, Julio, C. Rojas^1^, Mauricio Casas-Martínez^2^, Leopoldo Cruz-López^1^ and Edi A. Malo^1^


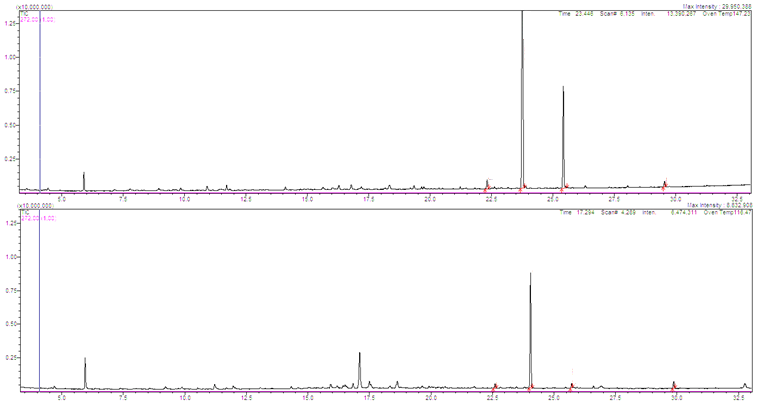


Phenol

4MP

4EP

Indolel

***Immature female rat extract***

***Immature male rat extract***

Phenole

4MP

4EP

Indole


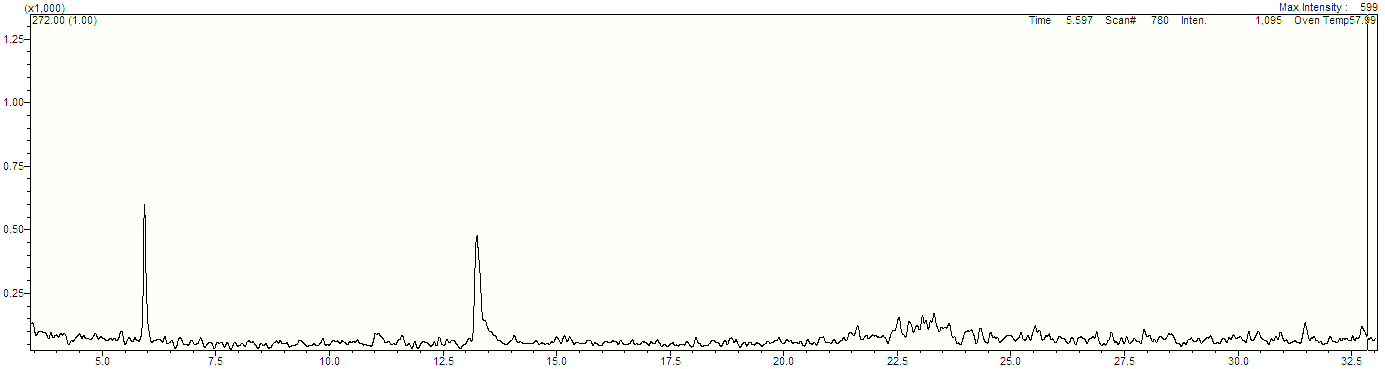


***Control***

Retention time

**Figure S1.** Gas chromatogram of the extracts of rat (female, male) and control in a GC-MS with a polar column. We found four peaks identified as phenol, 4-methylphenol, 4-ethylphenol, and indole.


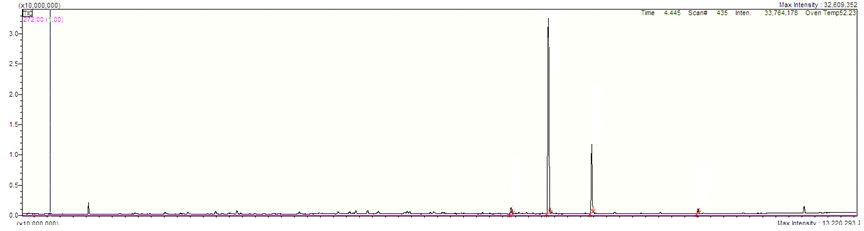


Phenol

4MP

4EP

Indole


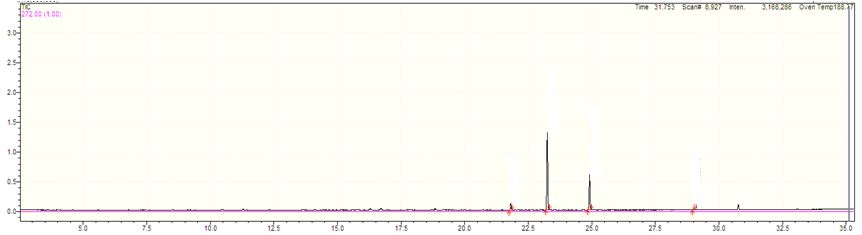


Phenol

4MP

4EP

Indole


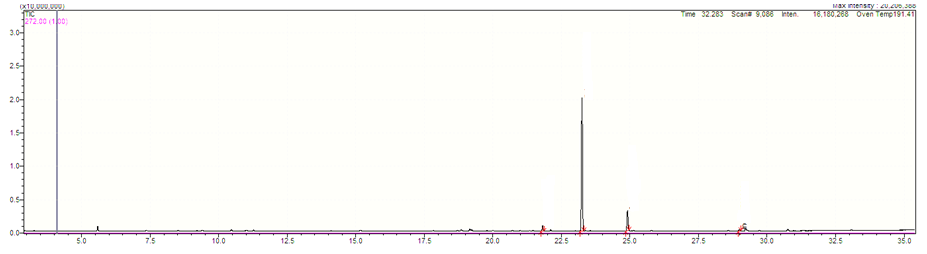


***Pregnant female rat extract***

Phenol

4MP

4EP

Indole


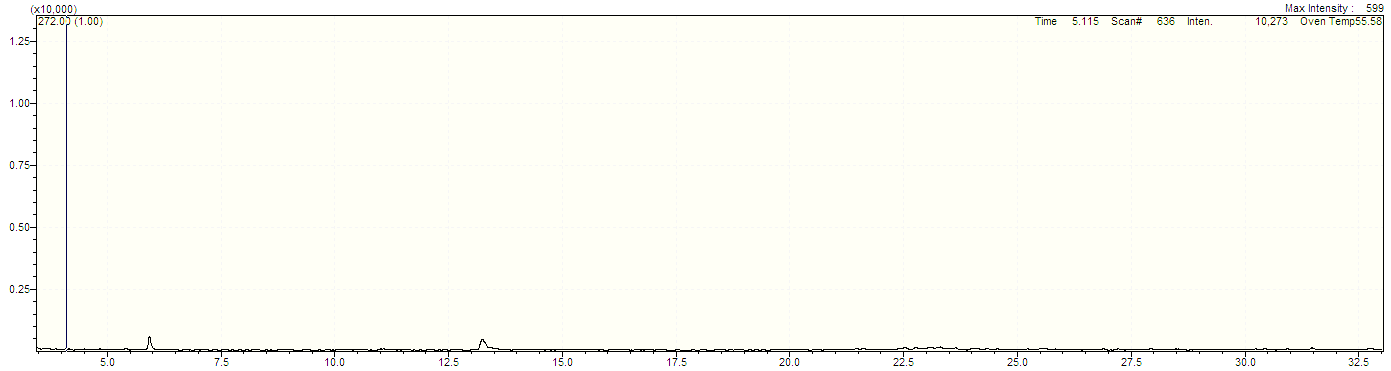


***Immature female rat extract***

***Mature female rat extract***

***Control***

Retention time

0

**Figure S2.** Gas chromatogram of the extracts of rat female (physiological stage) in a GC-MS with a polar column. We found four peaks identified as phenol, 4-methylphenol, 4-ethylphenol, and indole.

**Table S1.** Mean concentration (ng/µl ± SE) and proportion of volatiles identified in rat extracts obtained by dynamic headspace* Compared by sex and physiological stage.

**Sex**

**Physiological stage**

| **Comparison by** | **Physiological stage- Sex** | **Phenol (ng)** | **%** | **4-methylphenol (ng)** | **%** | **4-Ethylphenol (ng)** | **%** | **Indole (ng)** | **%** |
| --- | --- | --- | --- | --- | --- | --- | --- | --- | --- |
|  | Immature male | 2.9± 0.30 | 3.4 | 74.9± 6.13 | 87.7 | 2.8± 0.26 | 3.3 | 4.8± 0.49 | 5.6 |
|  | Immature female | 9.52 ± 1.14 | 1.8 | 394.7± 21.08 | 77.2 | 100.8± 10.16 | 19.7 | 6.5± 0.84 | 1.3 |
|  | Immature female | 9.52 ± 1.14 | 1.8 | 394.7± 21.08 | 77.2 | 100.8± 10.16 | 19.7 | 6.5± 0.84 | 1.3 |
|  | Mature female | 13.2± 2.31 | 5.1 | 202.2± 51.26 | 77.7 | 43 ± 8.88 | 16. 5 | 1.7±0.39 | 0.70 |
|  | Pregnant female | 9.4±1.45 | 2.8 | 240.8± 61.63 | 72. 5 | 73.6± 15.10 | 22.2 | 8.3± 2.66 | 2. 5 |

*This concentration was obtained by GC-FID using a calibration curve in ng/µl. N = 5 replicates.

**Table S2.** Release rate and load of synthetic compound in the rubber septum. The amounts used were according to the release rate of each compound, as well as the proportion in which each compound is found in the extract. The release rate was determined by weight loss of the rubber septum.

| Compound | 4-compound blend | 3-compound blend | 2-compound blend | Release rate  (mg/h) |
| --- | --- | --- | --- | --- |
| 4-methylphenol | 356.3 mg | 356.3 mg | 356.3 mg | 1.6 |
| 4-ethylphenol | 90.9 mg | 90.9 mg | 90.9 mg | 1.5 |
| Indole | 6.0 mg | 6.0 mg | ------- | 1.3 |
| Phenol | 8.3 mg | ------ | ------- | 1.4 |
